# PEDOT:PSS/graphene quantum dots films with enhanced thermoelectric properties via strong interfacial interaction and phase separation

**DOI:** 10.1038/s41598-018-24632-4

**Published:** 2018-04-24

**Authors:** Fei-Peng Du, Nan-Nan Cao, Yun-Fei Zhang, Ping Fu, Yan-Guang Wu, Zhi-Dong Lin, Run Shi, Abbas Amini, Chun Cheng

**Affiliations:** 1 School of Materials Science and Engineering, Wuhan Institute of Technology, Wuhan, 430074 China; 2 Department of Materials Science and Engineering, Southern University of Science and Technology, Shenzhen, 518055 China; 3 Center for Infrastructure Engineering, Western Sydney University, Kingswood, NSW 2751 Australia; 4 Department of Mechanical Engineering, Australian College of Kuwait, Mishref, Kuwait

## Abstract

The typical conductive polymer of PEDOT:PSS has recently attracted intensive attention in thermoelectric conversion because of its low cost and low thermal conductivity as well as high electrical conductivity. However, compared to inorganic counterparts, the relatively poor thermoelectric performance of PEDOT:PSS has greatly limited its development and high-tech applications. Here, we report a dramatic enhancement in the thermoelectric performance of PEDOT:PSS by constructing unique composite films with graphene quantum dots (GQDs). At room temperature, the electrical conductivity and Seebeck coefficient of PEDOT:PSS/GQDs reached to 7172 S/m and 14.6 μV/K, respectively, which are 30.99% and 113.2% higher than those of pristine PEDOT:PSS. As a result, the power factor of the optimized PEDOT:PSS/GQDs composite is 550% higher than that of pristine PEDOT:PSS. These significant improvements are attributed to the ordered alignment of PEDOT chains on the surface of GQDs, originated from the strong interfacial interaction between PEDOT:PSS and GQDs and the separation of PEDOT and PSS phases. This study evidently provides a promising route for PEDOT:PSS applied in high-efficiency thermoelectric conversion.

## Introduction

Thermoelectric materials have been developed and used in fine devices to assist reducing environmental pollution and energy crisis in 21^st^ century^1–3^. These materials have unique capability to directly convert heat to electric energy. The efficiency of this energy conversion is governed by the dimensionless figure-merit parameter Z in *ZT* = *S*^2^*σT*/*k*, where *S* is the Seebeck coefficient, *σ* is the electrical conductivity, *T* is the absolute temperature, and *k* is the thermal conductivity^3^. High performance thermoelectric materials require high Seebeck coefficient and electrical conductivity, as well as low thermal conductivity.

It is well-known that the thermoelectric applications of traditional inorganic semiconductor materials, such as Bi_2_Sb_3_, Bi_2_Te_3_ and PbTe, have little improvement due to high thermal conductivity and high cost^2,3^. Compared to inorganic thermoelectric materials, conducting polymers have shown specific advantages, such as low cost, light weight, good flexibility, rich resources, and low thermal conductivity in waste heat harvesting^4–6^. Among conducting polymers, commercially available poly3,4-ethylene-dioxythiophene:polystyrenesulphonate (PEDOT:PSS) possesses a great potential in thermoelectric applications due to its water-dispersibility, low cost, high transparency, and excellent processability^7–9^, nevertheless, the thermoelectric properties of this polymer is very low compared to its inorganic counterparts^10,11^. Many efforts, such as solvent post-treatment, redox state controlling and filler doping, have been made to enhance the thermoelectric properties of PEDOT:PSS. Effective solvents for post-treatment, including diethylene glycol (DEG), dimethyl sulfoxide (DMSO) and ethylene glycol (EG)^12–14^, can well-dissolve the PSS chains and remove PSS from the PEDOT:PSS structure, leading to close connection between the PEDOT particles and PEDOT:PSS with unique surface morphologies. As a result of selective removal of PSS (insulator, non-ionized dopant), the electrical conductivity of PEDOT:PSS can be greatly improved by the enhancement of charge transfer because of nearer distance between PEDOT chains^12,14^. Solvent post-treatment is simple, but mainly acts on the surface of samples and suffers from unstable performance. The redox state of PEDOT can be controlled to balance the Seebeck coefficient and electrical conductivity. For example, the redox state of PEDOT was adjusted via incorporating iron tosylate and tetrakis-(dimethylamino) ethylene (TDAE) instead of PSS; as a result of this, the electrical conductivity and Seebeck coefficient were well balanced, upgrading *ZT* to a high level of 0.25^15^. Nevertheless, the oxidation of PEDOT is hard to be controlled, and the properties of redox state PEDOT are not stable. Besides, highly conductive PEDOT:PSS still suffers from a low Seebeck coefficient^6^, which is generally measured in the range of 14–18 μV/K.

Highly conductive thermoelectric fillers have been used as dopants to increase the electrical conductivity to balance the power factor for greatly improving the thermoelectric properties of polymers, such as Ca_3_Co_4_O_9_^16^, Te-Bi_2_Te_3_^17^, Te^18^, graphene^19^, graphene oxide (GO)^20^, carbon nanotube^21^ and reduced graphene oxide (RGO)^22^. Among these fillers, graphene group has better potential for its outstanding carrier mobility, strong mechanical properties, large specific surface area, and excellent chemical tolerance^23–25^. Previous works have shown that graphene and its derivatives not only increase the electrical conductivity of conductive polymers, but also improve the Seebeck coefficient due to the energy filtering and ordered chains in the interfaces within the composite^26–31^. However, the power factor of PEDOT:PSS/graphene is still quite small compared with that of inorganic thermoelectric materials due to the low water-solubility and high aggregation of graphene in polymer matrix despite of its strong π-π interaction with PEDOT^26,28^. Theoretically, graphene in the shape of nanoribbons, antidots and nanorods, can obtain a higher ZT value after being tailored to a smaller size due to a lower thermal conductivity and stronger energy filtering effect^32,33^.

PEDOT:PSS/GQDs composites (GQDs stands for graphene quantum dots) have attracted great attentions in the photoelectric conversion field with prominent results^34,35^. Lim *et al*. represented the power conversion efficiency of the organic photovoltaic device containing the self-assembled PEDOT:PSS/GQDs organogel as the hole extraction layer was 26% higher than the device with pristine PEDOT:PSS^34^. Kepić *et al*. reported that PEDOT:PSS/GQDs films have lower sheet resistance and high transparency, which make PEDOT:PSS/GQDs good candidates for preparation of electrodes for optoelectronic device^35^. GQDs, with a size of several nanometers, have unique electronic properties, which can be easily prepared by chemical methods^36,37^. However, few efforts have been made on GQDs as fillers to be utilized in the thermoelectric filed. Besides, most PEDOT:PSS matrix used are commercial Clevios™ PH1000 containing 1 wt.% PEDOT: PSS with relative high Seebeck coefficient (about 15 μV/K) and high electricity (about 1000 S/cm). However, low viscosity of Clevios™ PH1000 (<50 cP) has limit its application in conducting inks for screen-printing where relatively high viscosity (∼10^3^ cP) are required to achieve good adhesion between the patterns and the substrate^38^. Here, we used PEDOT:PSS (screen-printing type) with 5 wt.% content instead of Clevios™ PH1000 because of its high viscosity, highly controllable film thickness, easy for spinning and ink-jet printing and potential application in flexible thermoelectric materials. However, the thermoelectric properties of PEDOT:PSS (5 wt.%) is very poor because of its low Seebeck coefficient (6.8 μV/K) and low electricity (about 55 S/cm). Therefore, we have carried out the research on the enhancement thermoelectric performance of the GQDs on the PEDOT:PSS (5 wt.%).

In this paper, we reported the significantly enhanced thermoelectric performance of PEDOT:PSS/GQDs thin films by using GQDs as fillers in the polymer matrix. The incorporation of GQDs into polymer matrix can adjust the PEDOT:PSS molecular structures via the electrostatic interaction between oxygen-containing group of GQDs and PSS, as well as the π-π conjugated interaction between PEDOT chains and graphene domains of GQDs. Notably, the electrical conductivity of PEDOT:PSS can be greatly increased, meanwhile the Seebeck coefficient can also be effectively improved due to the excellent energy filtering effect of GQDs.

## Results and Discussion

### Microstructure of GQDs

**Figure 1:**
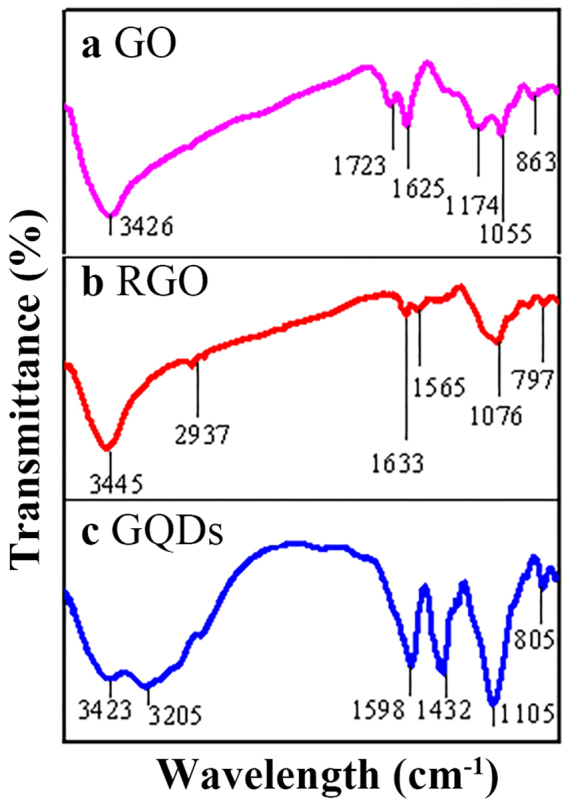
FTIR spectra of (**a**) GO, (**b**) RGO, and (**c**) GQDs.

Surface groups of GO, RGO and GQDs are characterized by FTIR spectra (Fig. 1). Figure 1a shows the vibration peaks of GO appearing at 3,426, 1,723 and 1,625 cm^−1^ attributed to plenty of hydroxyl groups (OH), carbonyl groups (C=O), and carboxylic acid groups (COOH), respectively. Additionally, the vibration peaks at 1,174 and 1,055 cm^−1^ are, respectively, attributed to the asymmetric and symmetric stretching vibrations of C-O-C bonds in the GO surface. Figure 1b demonstrates that the vibration peak of GO at 1723 cm^−1^ disappears in RGO and a new vibration peak appears at 2937 cm^−1^, indicating that the carbonyl groups (C=O) are reduced to methylene (-CH_2_-). Moreover, the intensity of the peak at 1625 cm^−1^ is depressed, suggesting that most of carbonyl groups are reduced. Figure 1c shows the FTIR spectra of GQDs, where the vibration peaks at 3423, 3205, 1598 and 1432 cm^−1^ correspond to the stretching vibration of O-H, N-H, C=O and C-N bonds, respectively. The existence of nitrogen-containing groups indicates the successfully doped GQDs by nitrogen atoms as described by others^39^.

**Figure 2:**
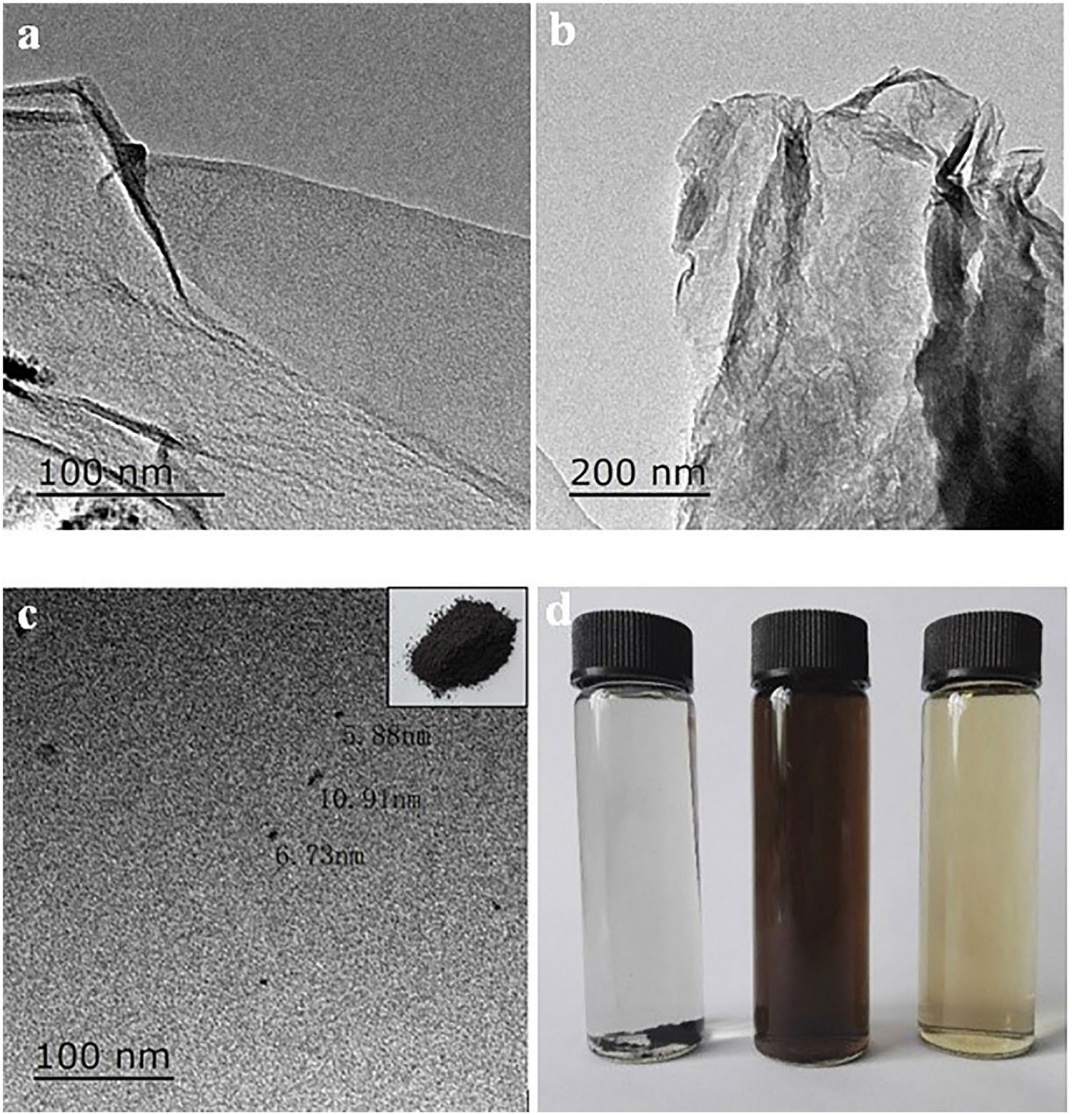
TEM images of (**a**) GO, (**b**) RGO, and (**c**) GQDs (inset image is GQDs powder). (**d**) their water-dispersing behavior (1 mg/mL) after an ultrasonic treatment for 30 days (left: RGO, middle: GO, right: GQDs).

Figure 2 shows a typical wrinkled sheet-shape structure for GO and RGO, while RGO sheets stack to form multilayered architectures by van der Waals force between the sheets. The TEM images verify well-dispersed GQDs with several nanometers in size and dot-like morphology. Thus, GQDs can be successfully prepared by tailoring GO. The inset optical image in Fig. 2c belongs to the as-prepared GQD powder, with a tan color similar to that of GO. As shown in Fig. 2d, GO, RGO and GQDs are dispersed into deionized water to form homogenous suspension liquids with a concentration of 1 mg/mL via an ultrasonic treatment. The dispersed suspension of GO and GQDs are quite stable, while RGO with a poor hydrophilicity is not well dispersed in water with a large amount of black precipitations appearing at the bottom of the vial. After a month, all RGO finally precipitated at the bottom of the vial, while GO and GQDs did not have significant precipitation. The excellent water-dispersion of GQDs is attributed to a large number of hydrophilic groups on their surface and boundary, such as -NH_2_ and -CONH_2_; this is confirmed by the functional groups observed from the above FTIR spectra.

### Microstructure of PEDOT:PSS-based films

**Figure 3:**
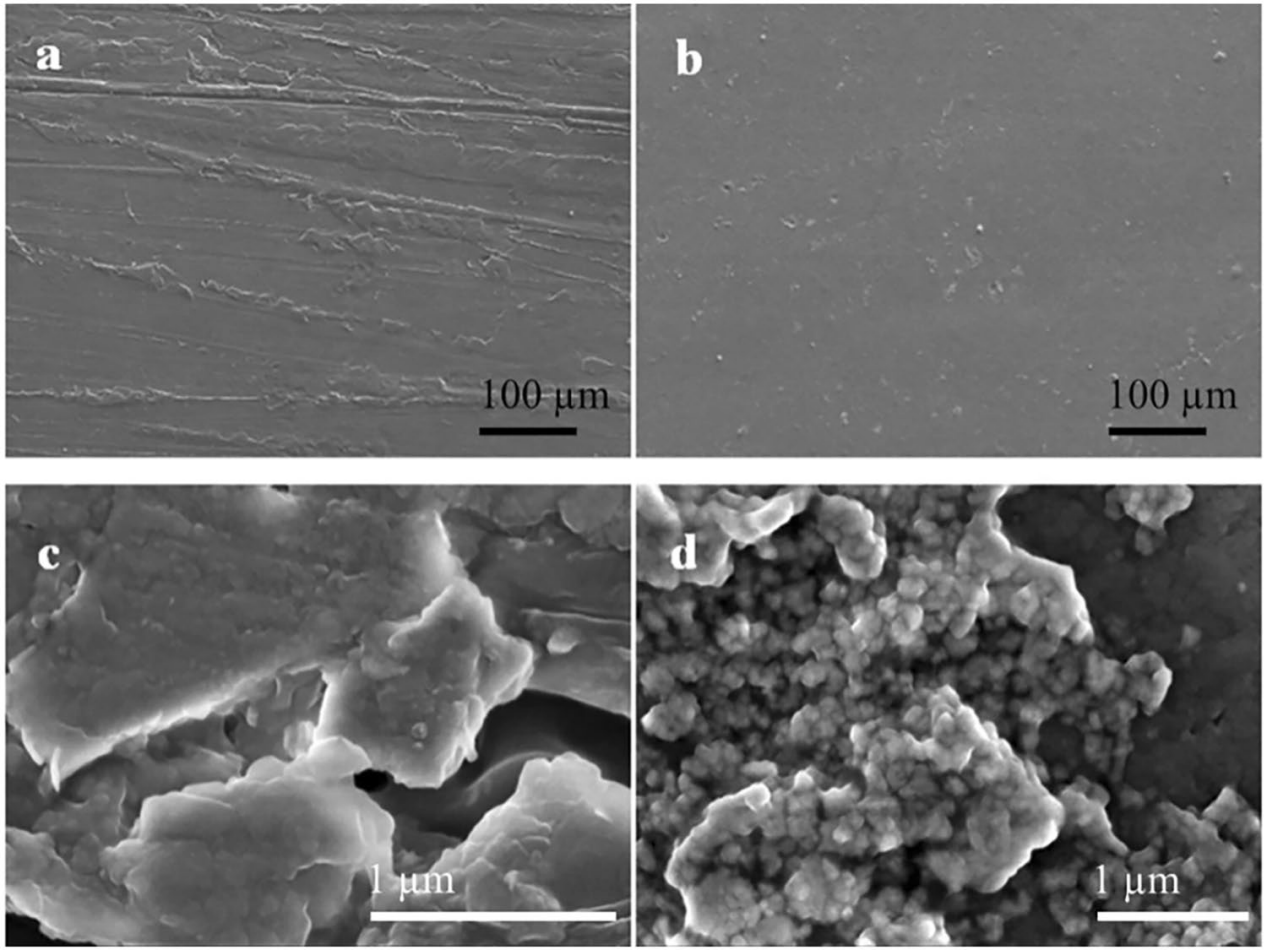
SEM images of PEDOT:PSS (**a**,**c**) and P-GQDs-10 (**b**,**d**).

SEM images demonstrate that all PEDOT:PSS-based films have relatively smooth and plain surface (Fig. 3a,b). However, the microstructures of their surface in high magnification have distinct difference with each other (Fig. 3c,d). The surface of PEDOT:PSS contains few grains with a good film-forming capability. Similar to PEDOT:PSS, the surface of P-RGO-10 is shown to be smooth and plain with few grains, while the surface of P-GO-10 is relatively rough and has few larger grains (Figure S1). After being doped by GQDs, the surface of P-GQDs-10 contains plenty of grains with uniform sizes. The variation of surface morphology of these three samples indicates the different interactions between polymer and different graphene derivatives. It is known that the PEDOT chains in the structure of PEDOT:PSS have plenty of π bonds. It is also well-known that RGO, GO and GQDs have abundant π bonds so that they can be well embedded into a PEDOT:PSS matrix by strong π-π interaction with PEDOT chains. Obviously, GO and GQDs possess plenty of hydrophilic groups, thus, they can not only build tight interaction with PEDOT chains due to π-π interaction, but also interact with PSS chains due to hydrophilic-hydrophilic interaction. These strong multiphase interactions possibly induce the phase separation between PEDOT chain and PSS chain, resulting in the grains formed on the surfaces of P-GO-10 and P-GQDs-10, as described in previous reports^40–42^.

The cross-section images depict different morphologies of PEDOT:PSS doped by different fillers (Figure S2). The PEDOT:PSS particles are connected with each other in the cross-section (Figure S2a), and, due to the evaporation of solvent, some voids are remained. Among the filler-doped PEDOT:PSS composites, P-GQDs-10 has the highest roughness and density in the cross-section, P-GO-10 comes to the second, while P-RGO-10 demonstrates the lowest roughness and density. These results are attributed to different types of interactions between graphene derivatives and polymers. RGO only has π-π interaction with PEDOT chains and weak interaction with PSS chains, whereas either GO or GQDs have strong interaction with both PEDOT chains and PSS chains, leading to the detachment in the PEDOT:PSS structure^34,35,40–42^.

**Figure 4:**
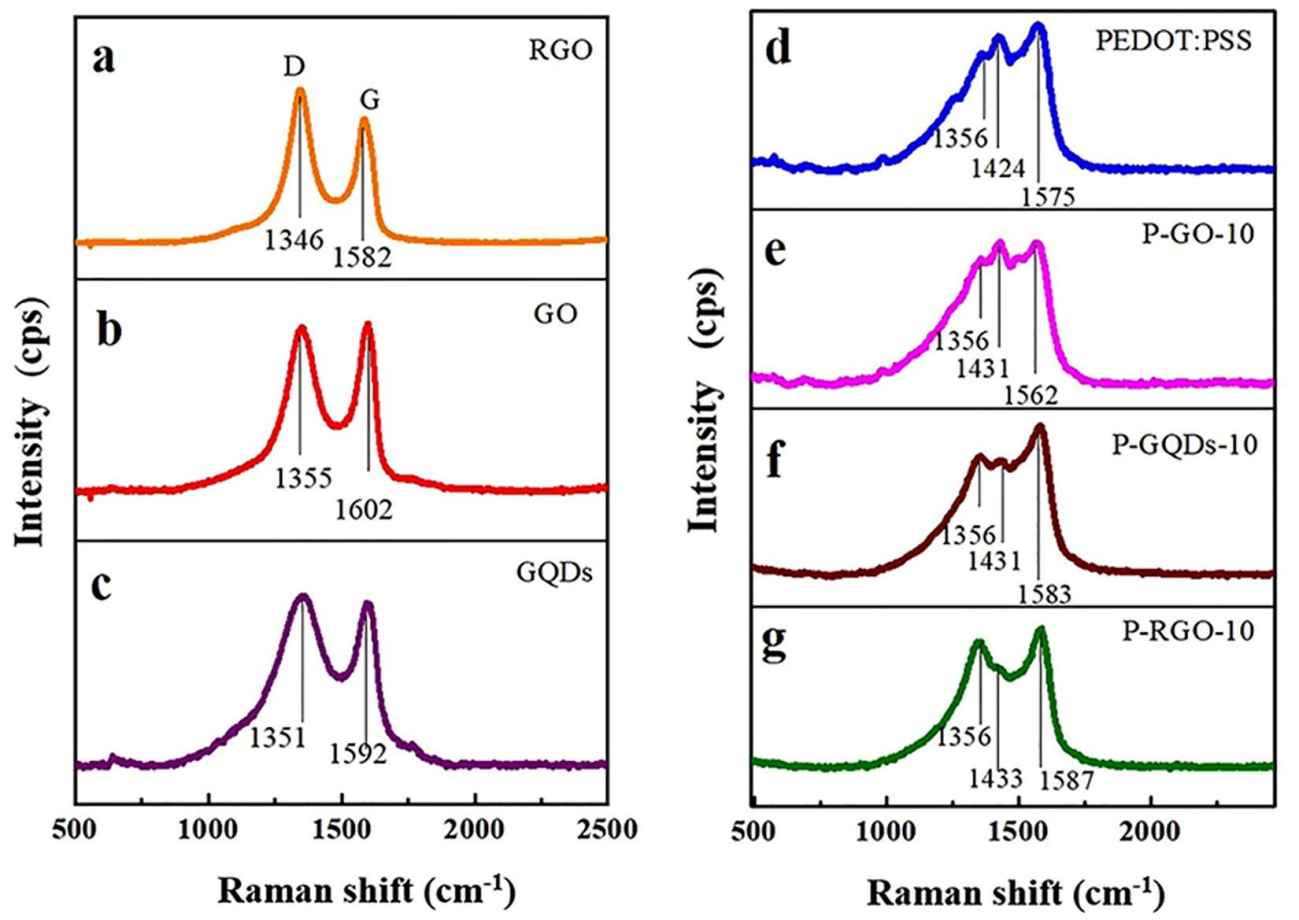
Raman spectra of (**a**) RGO, (**b**) GO, (**c**) GQDs, (**d**) PEDOT:PSS, (**e**) P-GO-10, (**f**) P-GQDs-10, and (**g**) P-RGO-10.

Raman spectra were used to reflect the defects and disordered structures of carbon-based materials. Raman spectra of RGO, GO, GQDs and their composites are shown in Fig. 4, where G peak can be attributed to the vibration of sp^2^ bonded carbon atoms, and D peak is corresponded to the vibration of carbon atoms with dangling bonds in carbon materials, which reflects the defects and the disordered structures in carbon-based materials^35^. D and G peaks of GO, RGO and GQDs are located at about 1350 and 1590 cm^−1^, respectively. Notably, all the D bands of RGO, GO and GQDs have almost the same intensity as G bands. The intensity ratio of D-band to G-band (I_D_/I_G_ ratio) is commonly used to characterize the defect quantity in graphitic materials^43^. The smaller ratio of I_D_/I_G_, the more sp^2^ hybrid carbon atoms, which is corresponding to the higher order degree of GO. I_D_/I_G_ of GO, RGO and GQDs are 1.23, 1.33, and 1.03, respectively, which are higher than the reported I_D_/I_G_ value^35,43,44^, indicating the existence of highly disorder structures in the as-prepared RGO, GO and GQDs.

From the Raman spectra of PEDOT:PSS, the corresponding characteristic peaks can be observed, such as the weak peaks of C-C inter-ring stretching (1269 cm^−1^), single C-C bond stretching (1369 cm^−1^), strong peaks of C=C symmetric stretching (1424 cm^−1^) and C=C antisymmetric stretching (1576 cm^−1^)^45^. Because of the interactions between graphene derivatives and PEDOT:PSS, all of the Raman peaks of doped PEDOT:PSS composites receive changes in intensity or position. The D band of all composites is located at ca. 1358 cm^−1^, demonstrating a shift towards a higher wavenumber than those of their corresponding fillers. The G band of P-RGO-10 (1588 cm^−1^) is shifted to a higher wavenumber compared to RGO, while the G bands of P-GO-10 (1567 cm^−1^) and P-GQDs-10 (1585 cm^−1^) are shifted to a lower wavenumber compared to those of the GO and GQDs, indicating GO and GQDs have strong interactions with unconjugated PSS. In addition, compared to the peak of pristine PEDOT:PSS at 1424 cm^−1^, the corresponding peaks for P-GO-10, P-GQDs-10 and P-RGO-10 are shifted to a higher wavenumber of ca. 1431 cm^−1^. This indicates the π-π interaction of aromatic structures of PEDOT and electron-rich GO, RGO and GQDs^26,28^. From the Raman spectra, it is concluded that GQDs have strong interaction both with PSS chains and PEDOT chains, which induces the phase separation of PEDOT:PSS and promotes the ordered alignment of PEDOT^46^.

**Figure 5:**
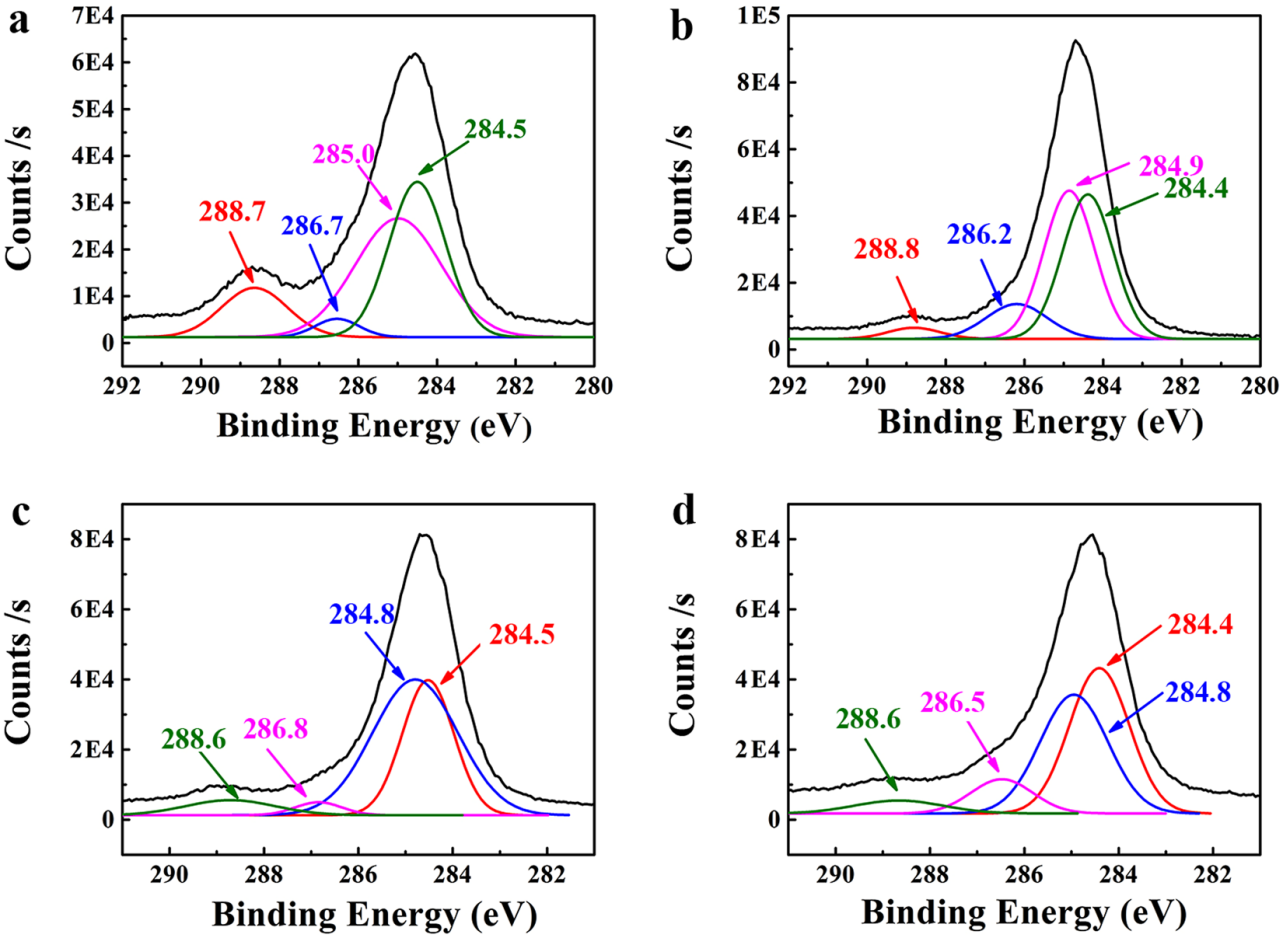
C(1 s) peak of XPS spectra of (**a**) PEDOT:PSS, (**b**) P-RGO-10, (**c**) P-GO-10, and (**d**) P-GQDs-10.

Surface chemical elements and structures of PEDOT:PSS-based composites were characterized by XPS. Typical carbon spectra (C1s) of PEDOT:PSS are demonstrated in Fig. 5a, including the aromatic C=C of PEDOT at 284.5 eV, the aliphatic C-C of PSS chains at 284.9 eV^47^, the C-O/C-S bonds at 286.7 eV, and the C=O/C=S bonds at 288.7eV^47,48^. The red shift of C=C bond (284.4 eV) after adding RGO and C-C bond (284.8 eV) after adding GO, indicates the strong π-π interaction between PEDOT chains and RGO, as well as a good compatibility between the hydrophilic group of GO and PSS chains, respectively. After adding GQDs, both the C=C bond and C-C bond have a red shift (Fig. 5d), indicating GQDs have a strong interaction with both PEDOT and PSS chains. The shift of C-O/C-S bond and C=O/C=S bonds further confirms the strong interaction.

**Figure 6:**
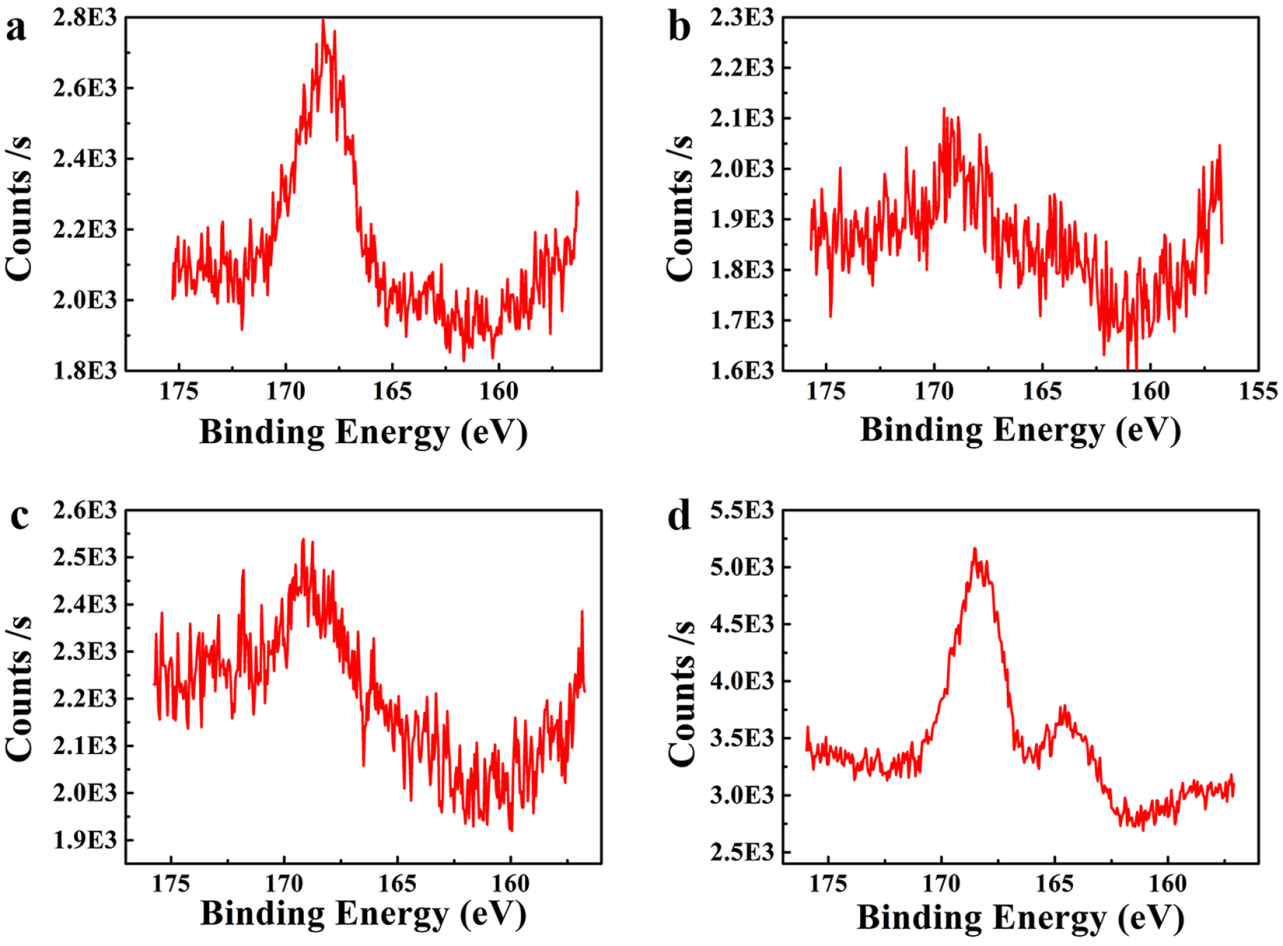
S(2p) peaks of XPS spectra of (**a**) PEDOT:PSS, (**b**) P-RGO-10, (**c**) P-GO-10, and (**d**) P-GQDs-10.

The two peaks in S(2p) spectra at 168–169 eV and 164–165 eV (Fig. 6), respectively, correspond to sulfur signals from the oxidized form of sulfonate (in PSS chains) and the thiophene (in PEDOT chains), respectively^48,49^. All the sulfur signals from PEDOT are significantly weak (Fig. 6a–c)^41^, which infers coating the PEDOT chains by enriched-PSS chains and covering the sulfur signals of PEDOT. The sulfur signals from PSS are also weakened after adding GO and RGO compared to pristine PEDOT:PSS. This might be due to fillers exposed on surface and big graphene domains disturbing the sulfur signals from PSS. In fact, the addition of GQDs can effectively strengthen the sulfur signals of both PEDOT and PSS chains, which means that PEDOT and PSS chains were well detached due to their strong interaction with GQDs.

**Figure 7:**
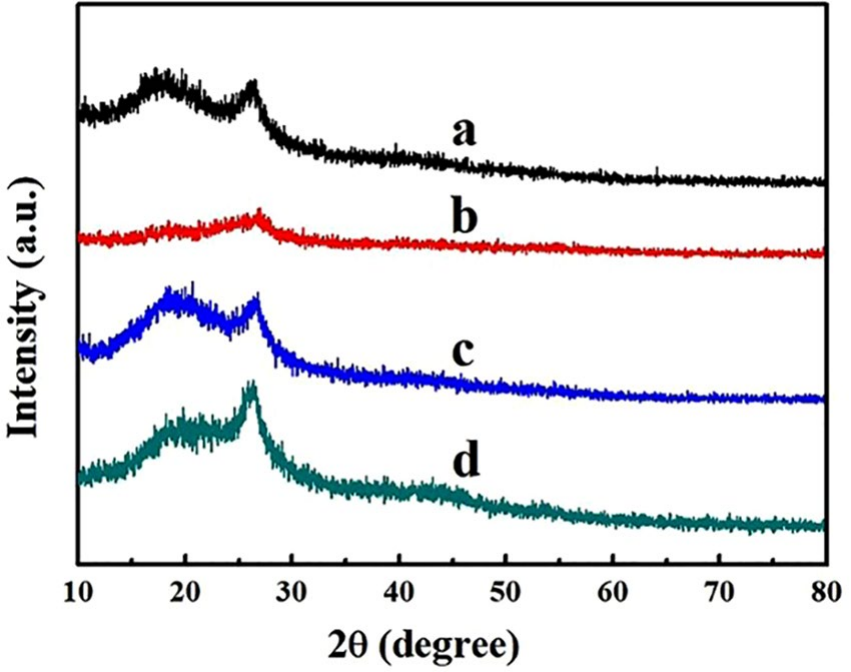
XRD patterns of (**a**) PEDOT: PSS, (**b**) P-RGO-10, (**c**) P-GO-10, and (**d**) P-GQDs-10.

The XRD patterns of PEDOT:PSS and their composites are shown in Fig. 7. The broad peak of PEDOT:PSS at 18.3° is corresponded to the enriched PSS chains structure^50^, while the peak at 26.2° is corresponded to the (020) plane of PEDOT:PSS^51,52^. However, the intensity of these two characteristic peaks become weak after adding a large amount of RGO which is due to hydrophobicity and aggregation of RGO. The interaction of aggregated RGO with PEDOT chains destroys the crystalline region and the stack structure of PEDOT:PSS. These two characteristic peaks are not affected after adding GO, indicating the uniform dispersion of GO in PEDOT:PSS matrix, which is caused by its strong interaction with PSS^53^. In addition, GQDs weakens the peaks at 18.2° and enhances the peak at 26.2°. This indicates the interaction between GQDs and PEDOT:PSS which induces the separation of PEDOT and PSS and creates the ordered alignment of PEDOT chains^54^.

### Thermoelectric properties of PEDOT:PSS-based composites

**Figure 8:**
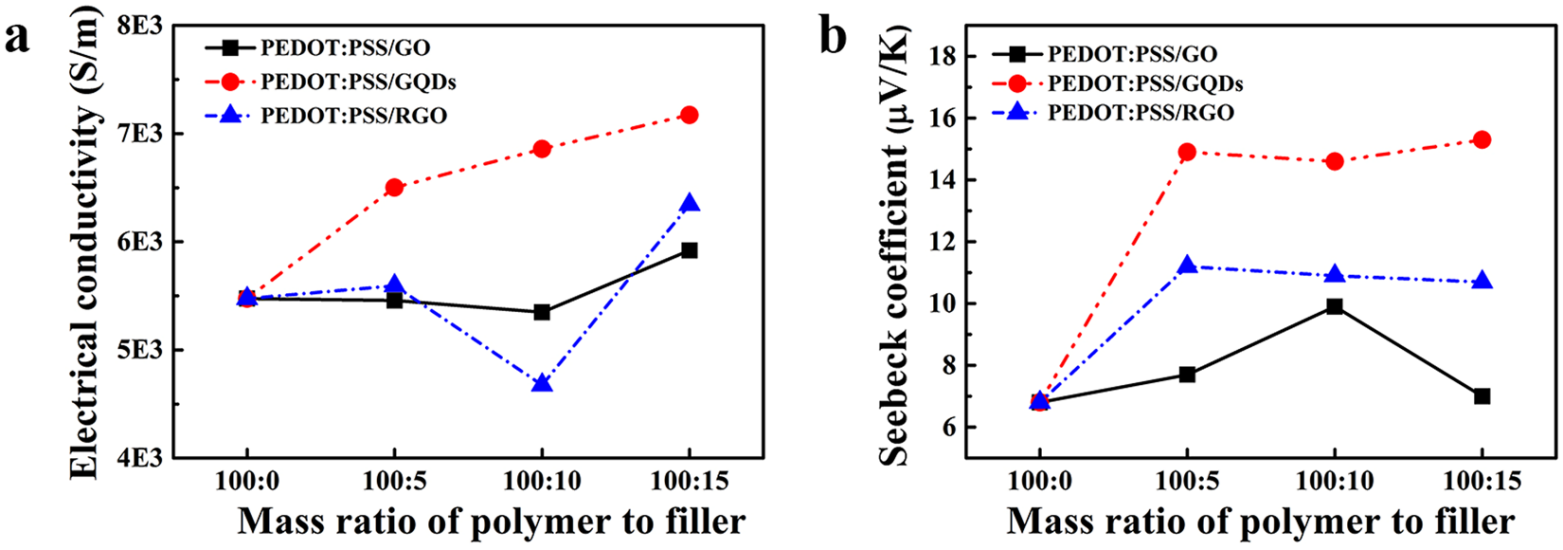
(**a**) Electrical conductivity and (**b**) Seebeck coefficient of PEDOT:PSS-based composites with different mass ratios of polymer to fillers at room temperature.

Figure 8 shows the electrical conductivity and Seebeck coefficient of PEDOT:PSS-based composites. Due to low electrical conductivity of GO, the electrical conductivity of PEDOT:PSS/GO increases slowly at higher GO contents. The electrical conductivity of PEDOT:PSS/RGO is even lower than that of pristine PEDOT:PSS when the mass ratio of polymer/fillers reaches to 100:10. This observation is attributed to the aggregation of RGO in the polymer matrix^28,55^. Noticeably, the addition of small amount of GQDs brings a great improvement on the electrical conductivity of PEDOT:PSS despite the low electrical conductivity of GQDs^56^ (Fig. 8a). When the mass ratio reaches 100:5 (P-GQDs-5), the electrical conductivity of PEDOT:PSS/GQDs increases from 5475 S/m of the pristine sample to 6489 S/m (Fig. 8a). Different interactions between graphene derivatives and polymers lead to different changes in the electrical conductivity of the corresponding composites. The improvement of electrical conductivity of PEDOT:PSS/GQDs based composites can be ascribed with the interaction between GQDs and PEDOT:PSS scaled to several nanometers. Because the size of PEDOT chains is equal to or even larger than that of GQDs, the as-formed conductive network mainly depends on the close connection among PEDOT chains, improving the electrical conductivity of the whole matrix. Figure 8b shows that all the composites have Seebeck coefficients higher than 6.8 μV/K of pristine PEDOT:PSS. The Seebeck coefficient of PEDOT:PSS/GQDs is the highest and reaches to 15 μV/K, the Seebeck coefficient of PEDOT:PSS/RGO is the moderate one and reaches to 11 μV/K, and the Seebeck coefficient of PEDOT:PSS/GO is close to that of pristine PEDOT:PSS and reaches ca.7~9 μV/K. Both the Seebeck coefficients of PEDOT:PSS/GO and PEDOT:PSS/RGO increase firstly and then decrease. This observation suggests weak carriers transport in the interface at the higher fillers contents, which is originated from inefficient connections between PEDOT chains and fillers^28^.

**Figure 9:**
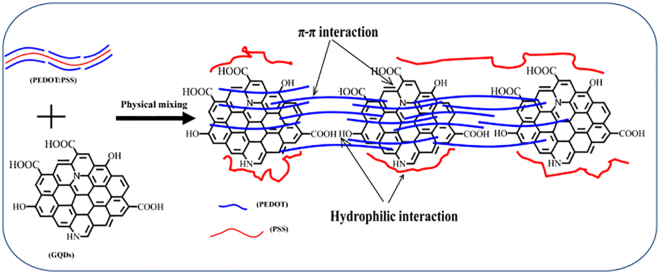
The scheme of assembly process of GQDs and PEDOT:PSS.

Compared to pristine PEDOT:PSS of 5 wt.%, the Seebeck coefficient of PEDOT:PSS/GQDs can be greatly enhanced by 125%. Zhang *et al*. have reported that nanostructured fillers in a PEDOT:PSS matrix can efficiently improve the Seebeck coefficient of the composites via the filtration of low-energy carriers and transporting high-energy carriers^57^; this is called energy-filtering effect. We believe the enhancement of Seebeck coefficient of the as-prepared composites also follows the same mechanism. Based on the above microstructures and thermoelectric properties, we propose a dual assembly structure to explain the formation of PEDOT:PSS/GQDs composites as shown in Fig. 9. The results of Raman, XPS, and XRD in this work give reliable evidences on the existence of strong interactions in GQDs with both PEDOT chains and PSS chains at a molecular level. The strong interactions lead to the phase separation between PEDOT and PSS^34,35^. Thus, GQDs not only connect PEDOT chains tightly as bridges, but make PEDOT chains orderly aligned on the surface of GQDs via π-π interaction, which improves the electrical conductivity of PEDOT:PSS. PSS chains is detached and curled around the edge of GQDs via the assembly of hydrophilic groups between GQDs and PSS (Fig. 9). The ordered assembly of PEDOT and GQDs provides the fast transporting of carriers and thus can improve the Seebeck coefficient. Meanwhile, the strong interaction under the molecular level in the interface of PEDOT:PSS and GQDs can provide energy filtering effect, further improving the Seebeck coefficient. This dual assembly can provide unique thermoelectric properties for PEDOT:PSS/GQDs with the highest Seebeck coefficient among three composites. Besides, we note that the similar enhanced effect of GQDs on Seebeck coefficient were observed in the commercial Clevious PH1000/GQDs, which contains 1 wt.% PEDOT: PSS. The Seebeck coefficient increases firstly at lower GQDs content and then decreases at higher GQDs content. This result suggests that GQDs effect on Seebeck coefficient is a general phenomenon (Figure S3) and this conclusion is also supported by the most recent work reported by Kim, S. H.^58,59^.

**Figure 10:**
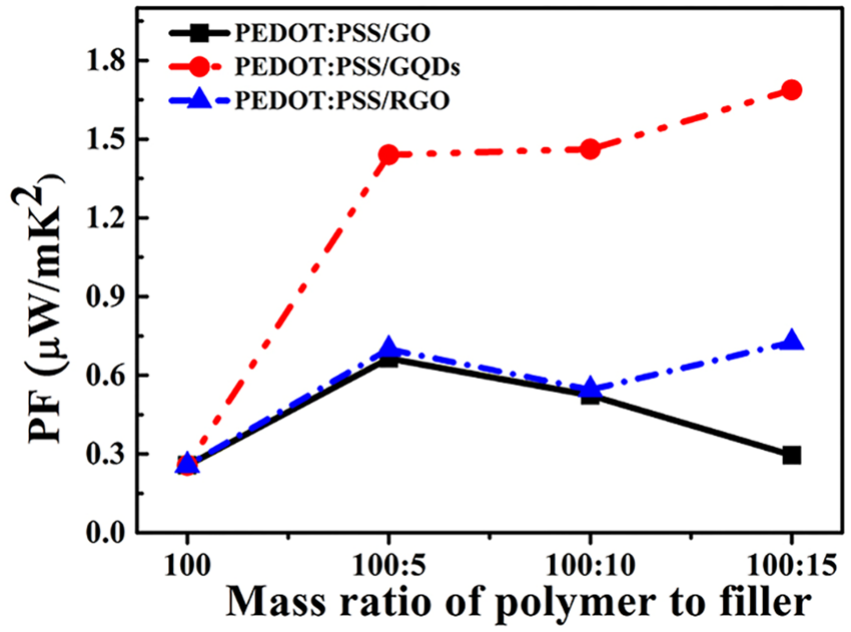
The power factor of the PEDOT:PSS-based composites versus mass ratio examined at room temperature.

Figure 10 compares the power factor of PEDOT:PSS-based composites at room temperature with different mass ratios. It is seen that PEDOT:PSS/GQDs composites with a properly high content of filler have much higher power factor than pristine PEDOT:PSS and the other two composites. The power factor of PEDOT:PSS/GQDs (P-GQDs-15) is increased by 550% compared to pristine PEDOT:PSS, which further confirms the strong interaction between GQDs and PEDOT:PSS/PSS chain. In this study, PEDOT:PSS in use was water solution with a high concentration of PEDOT:PSS (5 wt.%). High content of insulator PSS led to lower electrical conductivity of PEDOT:PSS in use than that of Clevios PH-1000, a high conductivity and commercial grade of PEDOT:PSS water solution at a low concentration of PEDOT:PSS (1.0–1.3 wt.%) often used in previous reports^27,28^. As a result, the electrical conductivity of as-prepared composites is quite low, and the obtained power factor is much lower than that of the composites with Clevios PH-1000 as polymer matrix^27,28^. However, the increase rate in the power factor of PEDOT:PSS caused by GQDs doping is significantly higher than those caused by the synergistic effect of GO and hydrazine treatment (331%)^60^, the synergistic effect of graphene and hydrazine treatment (500%)^28^, the synergistic effect of graphene and carbon nanotubes (108%)^61^, and other graphene based PEDOT:PSS^27^. In addition, PEDOT:PSS with high content of PSS possesses high viscosity which is necessary for conducting inks^38^. What’s more, PSS possesses sufficient sulfonate groups which enable uniform anchoring and good dispersion of various materials through electrostatic repulsion interactions^26^. Therefore, PSS is a promising candidate for tuning the viscosity of PEDOT:PSS solutions for screen-printing onto flexible plastic substrates and PEDOT:PSS with high content of PSS has potential application in flexible thermoelectric materials.

## Conclusions

PEDOT:PSS/GQDs composites were successfully prepared via simple casting methods. The strong interactions between GQDs and PEDOT chains via π-π bonding and between GQDs and PSS chains via hydrophilic groups led to decoupling and phase separation of PEDOT chains and PSS chains. The as-obtained microstructures (P-GQDs-10) simultaneously enhanced the electrical conductivity and Seebeck coefficient of PEDOT:PSS. The power factor of the optimized PEDOT:PSS/GQDs composite was increased by 550% compared to that of pristine PEDOT:PSS. This study provided a promising route for PEDOT:PSS applied in high-efficient thermoelectric conversion.

## Materials and Methods

### Materials

PEDOT:PSS solution (5.0 wt.%) was purchased from Aldrich Chemical Co., Inc. (USA). Graphite, sodium nitrate, and potassium permanganate were provided by Aladdin Industrial Co. (Shanghai, China). DMSO, EG, H_2_O_2_ (30%) and ammonia (25–28%) were obtained from Sinopharm Chemical Reagent Co., Ltd. (Beijing, China). All the chemicals were analytical grade and were directly used without further purification.

### Preparation of GQDs

GQDs were easily prepared via chemical cutting graphene oxide (GO) according to the previous report^37^. Firstly, GO was prepared according to Hummer’s method^62^, and described in our previous reports^63–65^. Then, 0.100 g GO was added to 10.0 mL distilled water and uniformly dispersed via one hour ultrasonic treatment. 200.0 mL of H_2_O_2_ (30%) and 50.0 mL of ammonia (25–28%) were added to the above GO stock solution. The mixture was kept at 90 °C for 24 hours with vigorous stirring. Rotary evaporation method was used to remove the residual H_2_O_2_, ammonia and water, and finally, GQDs were obtained via washing with ethanol and drying at 60 °C.

### Preparation of PEDOT:PSS/GQDs films

The PEDOT:PSS/GQDs films were prepared through a typical procedure as follows: a certain mass of GQDs were firstly added into 1.000 g PEDOT:PSS solution under an ultrasonic treatment for one hour. Afterwards, 500 μL EG/DMSO mixture (V:V, 3:1) was dropped into the PEDOT:PSS/GQDs dispersions. The as-obtained dispersions were stirred at room temperature for 24 hours. Finally, the PEDOT:PSS films were obtained with a length of 60 mm, a 20 width mm, and a thickness of 40 μm by casting the dispersions on the surface of glass plates and drying at 50 °C for 24 hours. The mass ratio of GQDs to PEDOT:PSS was selected as 5:100, 10:100, and 15:100, named after P-GQDs-5, P-GQDs-10 and P-GQDs-15, respectively. The reference experiments were done to obtain PEDOT:PSS/RGO films (named after P-RGO-5, P-RGO-10 and P-RGO-15) and PEDOT:PSS/GO films (named after P-GO-5, P-GO-10 and P-GO-15) using the same procedure with RGO and GO fillers, respectively.

### Characterization

The morphology of GO, RGO, and GQDs structures was examined by field-emission transmission electron microscope (FE-TEM, JEM-2100F). Additionally, the cross-fracture morphology and microstructure of the films were observed using a scanning electron microscope (SEM, JEOL JSM-6700F, Japan). The Raman spectra were measured by Raman spectrometer (DXR, USA) at an excitation wavelength of 532 nm. Fourier transform infrared (FTIR) spectra were recorded using Nicolet 6700 FTIR spectrometer (USA). X-ray photoelectron spectroscopy (XPS) was performed using a VG Multilab 2000 system with a monochromatic Mg-K_α_ source operated at 20 kV. X-ray diffraction (XRD) patterns at wide-angle (from 10° to 90° 2θ) and Cu-K_α_ radiation (λ = 0.15406 nm) were obtained using New D8-Advance/Bruker-AXS (Germany) powder X-ray diffractometer operating at 40 kV and 30 mA, a scanning rate of 6°/min and a scanning step of 0.02. The Seebeck coefficient and electrical conductivity were characterized at room temperature using a thermoelectric parameter test system (Namicro-III, Wuhan Schwab Instruments, China) with 7.5 mm probe spacing. All the samples had similar dimension of 14.50 mm × 14.50 mm × 40 μm [length (*l)* × width (*w)* × thickness (*d*)]. Five samples were characterized to obtain the average value. For measuring the Seebeck coefficient, the temperature gradient of collection points was set from 0.3 to 3.5 °C along the longitudinal direction of samples. The power factor (*PF*) was calculated from Equation (1):

1 $$PF={S}^{2}\sigma $$

where *S* is the Seebeck coefficient, *σ* is the electrical conductivity.

### Data availability

All data generated or analyzed during this study are included in this published article (and its Supplementary Information files).

## Electronic supplementary material

Supporting Information

## References

[1] Twaha S, Zhu J, Yan YY, Li B (2016). A comprehensive review of thermoelectric technology: Materials, applications, modelling and performance improvement. Renew. Sust. Energ. Rev..

[2] Tan GJ, Zhao LD, Kanatzidis MG (2016). Rationally designing high performance bulk thermoelectric materials. Chem. Rev..

[3] Zhao LD (2014). Ultralow thermal conductivity and high thermoelectric figure of merit in SnSe crystals. Nature.

[4] Hu XC, Chen GM, Wang X (2017). An unusual coral-like morphology for composites of poly(3,4-ethylenedioxythiophene)/carbon nanotube and the enhanced thermoelectric performance. Compos. Sci. Technol..

[5] Xu KL, Chen GM, Qiu D (2013). Convenient construction of poly(3,4-ethylenedioxythiophene)-graphene pie-like structure with enhanced thermoelectric performance. J. Mater. Chem. A.

[6] He M, Qiu F, Lin Z (2013). Towards high-performance polymer-based thermoelectric materials. Energy Environ. Sci..

[7] Yoo D, Kim J, Kim JH (2014). Direct synthesis of highly conductive poly(3,4-ethylenedioxythiophene):poly(4-styrenesulfonate) (PEDOT:PSS)/graphene composites and their applications in energy harvesting systems. Nano Res..

[8] Luo JJ (2013). Enhancement of the thermoelectric properties of PEDOT:PSS thin films by post-treatment. J. Mater. Chem. A.

[9] Yee SK, Coates NE, Majumdar A, Urban JJ, Segalman RA (2013). Thermoelectric power factor optimization in PEDOT:PSS tellurium nanowire hybrid composites. Phys. Chem. Chem. Phys..

[10] Du Y, Cai KF, Chen S, Cizek P, Lin T (2014). Facile preparation and thermoelectric properties of Bi2Te3 based alloy nanosheet/PEDOT:PSS composite films. ACS Appl. Mater. Interfaces.

[11] Zhang B, Sun J, Katz HE, Fang F, Opila RL (2010). Promising thermoelectric properties of commercial PEDOT:PSS materials and their Bi2Te3 powder composites. ACS Appl. Mater. Interfaces.

[12] Kim GH, Shao L, Zhang K, Pipe KP (2013). Engineered doping of organic semiconductors for enhanced thermoelectric efficiency. Nat. Mater..

[13] Xia Y, Ouyang J (2011). PEDOT:PSS films with significantly enhanced conductivities induced by preferential solvation with cosolvents and their application in polymer photovoltaic cells. J. Mater. Chem..

[14] Mengistie DA (2015). Enhanced thermoelectric performance of PEDOT:PSS flexible bulky papers by treatment with secondary dopants. ACS Appl. Mater. Interfaces.

[15] Bubnova O (2011). Optimization of the thermoelectric figure of merit in the conducting polymer poly(3,4-ethylenedioxythiophene). Nat. Mater..

[16] Liu CC (2011). Free-standing PEDOT-PSS/Ca_3_Co_4_O_9_ composite films as novel thermoelectric materials. J. Electron. Mater..

[17] Bae EJ, Kang YH, Jang KS, Lee C, Cho SY (2016). Solution synthesis of telluride-based nano-barbell structures coated with PEDOT:PSS for spray-printed thermoelectric generators. Nanoscale.

[18] Sahu A (2017). Bottom-up design of de novo thermoelectric hybrid materials using chalcogenide resurfacing. J. Mater. Chem. A.

[19] Chen Y (2015). PEDOT:PSS/graphene/PEDOT ternary film for high performance electrochemical electrode. J. Mater. Sci.: Mater. Electron.

[20] Yu JC (2014). Highly efficient polymer-based optoelectronic devices using PEDOT:PSS and a GO composite layer as a hole transport layer. ACS Appl. Mater. Interfaces.

[21] Wang WJ (2015). Effect of methanol addition on the resistivity and morphology of PEDOT:PSS layers on top of carbon nanotubes for use as flexible electrodes. ACS Appl. Mater. Interfaces.

[22] Jo K (2011). Stable aqueous dispersion of reduced graphene nanosheets via non-covalent functionalization with conducting polymers and application in transparent electrodes. Langmuir.

[23] Novoselov KS (2004). Electric field effect in atomically thin carbon films. Science.

[24] Geim AK (2009). Graphene: Status and Prospects. Science.

[25] Zhang LL, Zhou R, Zhao XS (2010). Graphene-based materials as supercapacitor electrodes. J. Mater. Chem..

[26] Kim GH, Hwang DH, Woo SI (2012). Thermoelectric properties of nanocomposite thin films prepared with poly(3,4-ethylenedioxythiophene) poly(styrenesulfonate) and graphene. Phys. Chem. Chem. Phys..

[27] Li F, Cai K, Shen S, Chen S (2014). Preparation and thermoelectric properties of reduced graphene oxide/PEDOT:PSS composite films. Synth. Met..

[28] Xiong JH (2015). Liquid exfoliated graphene as dopant for improving the thermoelectric power factor of conductive PEDOT:PSS nanofilm with hydrazine treatment. ACS Appl. Mater. Interfaces.

[29] Chen GM, Xu W, Zhu DB (2017). Recent advances in organic polymer thermoelectric composites. J. Mater. Chem. C.

[30] Li X, Liang L, Yang M, Chen G, Guo CY (2016). Poly(3,4-ethylenedioxythiophene)/graphene/carbon nanotube ternary composites with improved thermoelectric performance. Org. Electron.

[31] Gao C, Chen G (2016). Conducting polymer/carbon particle thermoelectric composites: Emerging green energy materials. Compos. Sci. Technol..

[32] Zhou S, Guo Y, Zhao J (2016). Enhanced thermoelectric properties of graphene oxide patterned by nanoroads. Phys. Chem. Chem. Phys..

[33] Yan Y, Liang QF, Zhao H, Wu CQ, Li B (2012). Thermoelectric properties of one-dimensional graphene antidot arrays. Phys. Lett. A.

[34] Lim HC (2015). Self-assembled poly(3,4-ethylene dioxythiophene):poly(styrenesulfonate)/ graphene quantum dot organogels for efficient charge transport in photovoltaic devices. ACS Appl. Mater. Interfaces.

[35] Kepić DP (2014). Preparation of PEDOT:PSS thin films doped with graphene and graphene quantum dots. Synth. Met..

[36] Li Y (2016). Chemical nature of redox-controlled photoluminescence of graphene quantum dots by post-synthesis treatment. J. Phys. Chem. C.

[37] Jiang F (2013). Eco-friendly synthesis of size-controllable amine-functionalized graphene quantum dots with antimycoplasma properties. Nanoscale.

[38] Cho S, Kim M, Jang J (2015). Screen-printable and flexible RuO2 nanoparticle-decorated PEDOT:PSS/graphene nanocomposite with enhanced electrical and electrochemical performances for high-capacity supercapacitor. ACS Appl. Mater. Interfaces.

[39] Dong YQ (2013). Carbon-Based Dots Co-doped with Nitrogen and Sulfur for High Quantum Yield and Excitation-Independent Emission. Angew. Chem. Int. Ed..

[40] Wu XK (2014). Highly conductive and uniform graphene oxide modified PEDOT:PSS electrodes for ITO-Free organic light emitting diodes. J. Mater. Chem. C.

[41] Raj PG, Rani VS, Kanwat A, Jang J (2016). Enhanced organic photovoltaic properties via structural modifications in PEDOT:PSS due to graphene oxide doping. Mater. Res. Bull..

[42] Dehsari HS, Shalamzari EK, Gavgani JN, Taromi FA, Ghanbary S (2014). Efficient preparation of ultralarge graphene oxide using a PEDOT:PSS/GO composite layer as hole transport layer in polymer-based optoelectronic devices. RSC Adv..

[43] Gupta A, Chen G, Joshi P, Tadigadapa S, Eklund PC (2006). Raman scattering from high-frequency phonons in supported n-graphene layer films. Nano Lett..

[44] Pimenta MA (2007). Studying disorder in graphite-based systems by Raman spectroscopy. Phys. Chem. Chem. Phys..

[45] Jiang XY (2017). High performance silicon–organic hybrid solar cells via improving conductivity of PEDOT:PSS with reduced graphene oxide. Appl. Surf. Sci..

[46] Seol YG, Trung TQ, Yoon OJ, Sohn IY, Lee NE (2012). Nanocomposites of reduced graphene oxide nanosheets and conducting polymer for stretchable transparent conducting electrodes. J. Mater. Chem..

[47] Guo X, Jian J, Lin L, Zhu H, Zhu S (2013). O_2_ plasma-functionalized SWCNTs and PEDOT/PSS composite film assembled by dielectrophoresis for ultrasensitive trimethylamine gas sensor. Analyst.

[48] Merche D (2010). One step polymerization of sulfonated polystyrene films in a dielectric barrier discharge. Plasma Processes Polym..

[49] Xia Y, Sun K, Ouyang J (2012). Solution-processed metallic conducting polymer films as transparent electrode of optoelectronic devices. Adv. Mater..

[50] Chen CH (2011). Mechanical characterizations of cast poly(3,4-ethylenedioxythiophene):poly(styrenesulfonate)/polyvinyl alcohol thin films. Synth. Met..

[51] Hu X, Chen G, Wang X, Wang H (2015). Tuning thermoelectric performance by nanostructure evolution of a conducting polymer. J. Mater. Chem. A.

[52] Zhao J, Tan DX, Chen GM (2017). A strategy to improve the thermoelectric performance of conducting polymer nanostructures. J. Mater. Chem. C.

[53] Giuri A (2017). Rheological and physical characterization of PEDOT: PSS/graphene oxide nanocomposites for perovskite solar cells. Polym. Eng. Sci..

[54] Lee CP (2017). A paper-based electrode using a graphene dot/PEDOT:PSS composite for flexible solar cells. Nano Energy.

[55] Hsu CT (2015). Synthesis and characterization of nano silver-modified graphene/PEDOT: PSS for highly conductive and transparent nanocomposite films. J. Polym. Res..

[56] Li XM, Rui MC, Song JZ, Shen ZH, Zeng HB (2015). Carbon and graphene quantum dots for optoelectronic and energy devices: a review. Adv. Funct. Mater..

[57] Zhang K (2015). Thermoelectric performance of p-type nanohybrids filled polymer composites. Nano Energy.

[58] Kim SH (2017). Control of the charge carrier concentration and hall mobility in PEDOT:PSS thermoelectric films. B. Kor. Chem. Soc..

[59] Xiong JH (2015). Liquid exfoliated graphene as dopant for improving thermoelectric power factor of conductive PEDOT:PSS nanofilm with hydrazine treatment. ACS Appl. Mater. Interfaces.

[60] Sarabia-Riquelme R, Ramos-Fernandez G, Martin-Gullon I, Weisenberger MC (2016). Synergistic effect of graphene oxide and wet-chemical hydrazine/deionized water solution treatment on the thermoelectric properties of PEDOT:PSS sprayed films. Synth. Met..

[61] Yoo D (2015). Effects of one- and two-dimensional carbon hybridization of PEDOT:PSS on the power factor of polymer thermoelectric energy conversion devices. J. Mater. Chem. A.

[62] Hummers WS, Offeman RE (1958). Preparation of graphitic oxide. J. Am. Chem. Soc..

[63] Du FP (2012). Water-soluble graphene grafted by poly (sodium 4-styrenesulfonate) for enhancement of electric capacitance. Nanotechnology.

[64] Du FP (2013). Enhanced electrochemical capacitance of polyaniline/graphene hybrid nanosheets with graphene as templates. Composites Part B.

[65] Du FP (2015). Enhancing the heat transfer efficiency in graphene–epoxy nanocomposites using a magnesium oxide–graphene hybrid structure. ACS Appl. Mater. Interfaces.

