# Supplementary material for: PEDOT:PSS/graphene quantum dots films with enhanced thermoelectric properties via strong interfacial interaction and phase separation
Source: Sci Rep. 2018 Apr 24;8:6441. doi: 10.1038/s41598-018-24632-4 (PMC5915444; doi:10.1038/s41598-018-24632-4)
Supplement: Supplementary file 1 — Supporting Information [file 41598_2018_24632_MOESM1_ESM.doc]

**Supporting Information for**

**PEDOT: PSS/Graphene Quantum Dots Films with Enhanced Thermoelectric Properties via Strong Interfacial Interaction and Phase Separation**

**Fei-Peng Du^1^, Nan-Nan Cao^1^, Yun-Fei Zhang^1^, Ping Fu^1^, Yan-Guang Wu^1^, Zhi-Dong Lin^1^, Run Shi^2^, Abbas Amini^3, 4^，Chun Cheng^2*^**

^1^School of Materials Science and Engineering, Wuhan Institute of Technology, Wuhan 430074, China

^2^Department of Materials Science and Engineering, Southern University of Science and Technology, Shenzhen 518055, China

^3^Center for Infrastructure Engineering, Western Sydney University, Kingswood, NSW 2751, Australia

^4^Department of Mechanical Engineering, Australian College of Kuwait, Mishref, Kuwait

***Corresponding author:** [chengc@sustc.edu.cn](mailto:chengc@sustc.edu.cn)

**
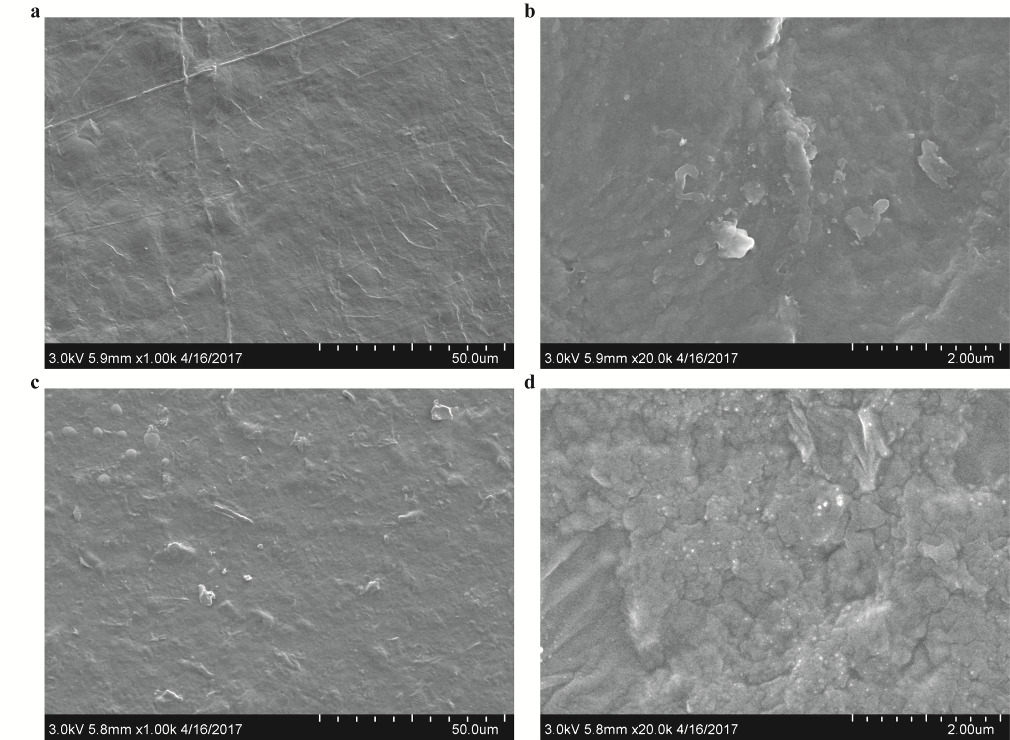
**

**Figure S1.** Surface SEM images of (a) P-RGO-10 and (b) its magnified version, as well as SEM images of (c) P-GO-10 and (d) its magnified version.

**
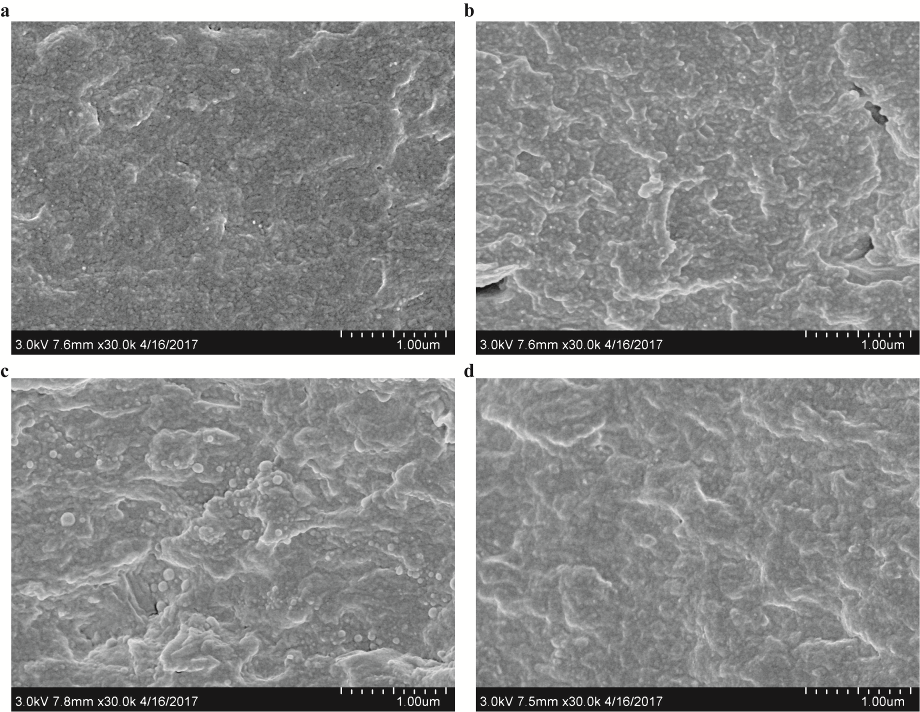
**

**c**

**Figure S2.** Cross-section SEM images of (a) PEDOT: PSS, (b) P-RGO-10, (c) P-GO-10 and (d) P-GQDs-10.


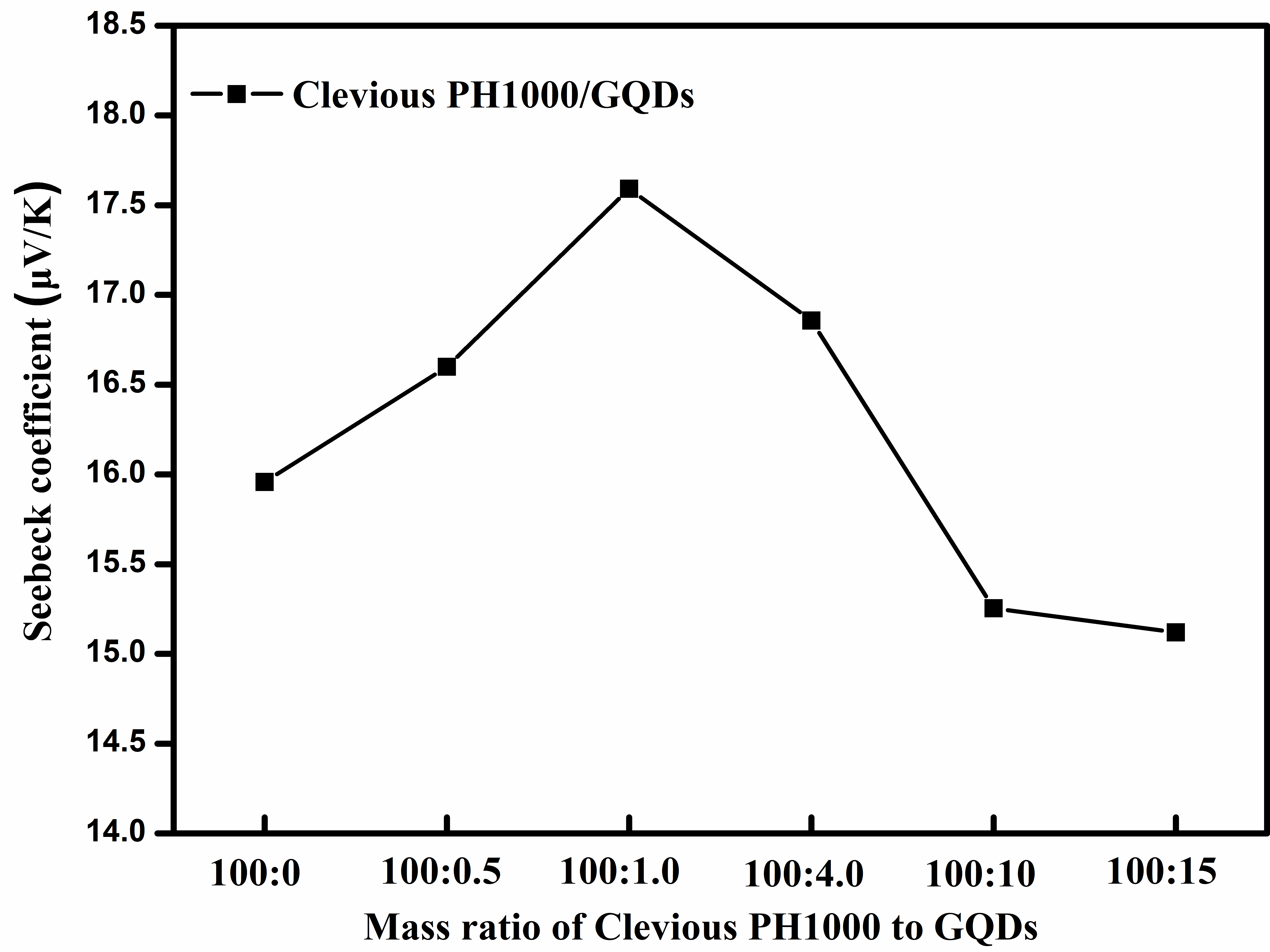


**Figure S3.** Seebeck coefficient of Clevious PH1000/GQDs composites with different mass ratios of Clevious PH1000 to GQDs at room temperature.
